# Fitness Features Involved in the Biocontrol Interaction of *Pseudomonas chlororaphis* With Host Plants: The Case Study of PcPCL1606

**DOI:** 10.3389/fmicb.2019.00719

**Published:** 2019-04-10

**Authors:** Eva Arrebola, Sandra Tienda, Carmen Vida, Antonio de Vicente, Francisco M. Cazorla

**Affiliations:** ^1^Departamento de Microbiología, Facultad de Ciencias, Universidad de Málaga, Málaga, Spain; ^2^Instituto de Hortofruticultura Subtropical y Mediterránea “La Mayora” IHSM, UMA-CSIC, Málaga, Spain

**Keywords:** *Pseudomonas chlororaphis*, root colonization, biocontrol, avocado, antifungals

## Abstract

The goal of this mini review is to summarize the relevant contribution of some beneficial traits to the behavior of the species *Pseudomonas chlororaphis*, and using that information, to give a practical point of view using the model biocontrol strain *P. chlororaphis* PCL1606 (PcPCL1606). Among the group of plant-beneficial rhizobacteria, *P. chlororaphis* has emerged as a plant- and soil-related bacterium that is mainly known because of its biological control of phytopathogenic fungi. Many traits have been reported to be crucial during the multitrophic interaction involving the plant, the fungal pathogen and the soil environment. To explore the different biocontrol-related traits, the biocontrol rhizobacterium PcPCL1606 has been used as a model in recent studies. This bacterium is antagonistic to many phytopathogenic fungi and displays effective biocontrol against fungal phytopathogens. Antagonistic and biocontrol activities are directly related to the production of the compound 2-hexyl, 5-propyl resorcinol (HPR), despite the production of other antifungal compounds. Furthermore, PcPCL1606 has displayed additional traits regarding its fitness in soil and plant root environments such as soil survival, efficient plant root colonization, cell-to-cell interaction or promotion of plant growth.

## Introduction

Since the earliest studies, soil has been described as an infinite source of microorganisms with beneficial activities that promote plant health (Waksman and Woodruff, 1940). Inside the soil, the rhizosphere environment is considered the soil-plant root interphase where potentially beneficial rhizobacteria are established. The plant-beneficial microbial life can be actively recruited by the plant rhizosphere (Berendsen et al., 2018) and can finally result in the biological control of the disease (Babalola, 2010). These biocontrol rhizobacteria can use a wide range of mechanisms involved in the suppression of plant pathogens. A diverse range of bacterial genera, such as *Bacillus*, *Pseudomonas*, *Serratia*, *Stenotrophomonas*, and *Streptomyces*, has been commonly described asx beneficial rhizobacteria (Berg, 2009). Among them, representatives of the *Pseudomonas* genus have been commonly associated with the rhizosphere and soil habitats (Lugtenberg and Dekkers, 1999). This bacterial genus has also been widely studied due to its ability to produce antifungal compounds, compete for niche and/or nutrients on the rhizosphere, and elicit induced systemic resistance in plants (Haas and Défago, 2005). Currently, many strains belonging to the group of fluorescent *Pseudomonas* are known to enhance plant growth promotion and reduce the severity of various diseases (Ganeshan and Kumar, 2005; Mercado-Blanco and Bakker, 2007; Weller, 2007).

The *Pseudomonas fluorescens* complex is one of the most diverse bacterial groups within the *Pseudomonas* genus and comprises more than fifty validly named species and many unclassified isolates (Garrido-Sanz et al., 2017). Many strains of this complex have been isolated from plant-related environments, and several species can be considered beneficial since many are described as plant growth-promoting rhizobacteria and/or minimize the effects of phytopathogens (PGPR; Kang et al., 2006; Raaijmakers et al., 2009). The beneficial effects displayed by some bacteria result from the expression of multiple activities that act directly and indirectly inhibiting pathogen activities and promoting plant health (McSpadden, 2007). To date, a number of studies have characterized the environmental factors that affect the abundance of different pseudomonad populations below ground (Berg et al., 2002; Ownley et al., 2003; Mazzola et al., 2004; Bergsma-Vlami et al., 2005). *Pseudomonas* species most commonly reported to include plant beneficial rhizospheric strains are *Pseudomonas aureofaciens*, *Pseudomonas brassicacearum*, *Pseudomonas chlororaphis*, *P. fluorescens*, *Pseudomonas Protegens*, and *Pseudomonas putida*.

## Beneficial Traits of Rhizospheric *Pseudomonas chlororaphis* Strains

Among the beneficial *Pseudomonas* spp., *P. chlororaphis* has evolved to be a common inhabitant of the root environment of many plants. Moreover, it has been extensively reported the role of specific traits that render this bacterium able to be used as an inoculant for biofertilization, phytostimulation, and biocontrol purposes (Bloemberg and Lugtenberg, 2001).

### Subspecies of *Pseudomonas chlororaphis*

*Pseudomonas chlororaphis*, *P. aureofaciens*, and *Pseudomonas aurantiaca* were initially included in the Approved List of Bacterial Names (Skerman et al., 1980) and considered separate species in the first edition of *Bergey’s Manual of Systematic Bacteriology* by Palleroni (1984). However, the results obtained by Peix et al. (2007) of fatty acid analysis, phenotypic characterization, 16S rRNA gene sequencing, and DNA-DNA relatedness together with the results obtained by Hilario et al. (2004) on the phylogenetic analysis of several housekeeping genes, support the reclassification of *P. aurantiaca* as a later heterotypic synonym of *P. chlororaphis*. The results published by Peix et al. (2007) also reveal that strains of *P. aurantiaca*, *P. aureofaciens*, and *P. chlororaphis* form three clearly distinguishable groups within *P. chlororaphis* that merit the status of subspecies. Therefore, the current classification is *P. chlororaphis* subsp. *chlororaphis* subsp. nov., *P. chlororaphis* subsp. *aureofaciens* subsp nov., comb. nov. and *P. chlororaphis* subsp. *aurantiaca* subsp. nov., comb. nov. (Peix et al., 2007). Three years later, Burr et al. (2010) added a new subspecies to *P. chlororaphis* and placed it on a distinct branch within this species with the name *P. chlororaphis* subsp. *piscium* subsp. nov. The current reports of sequenced bacterial genomes of *P. chlororaphis* strains (Calderón et al., 2015; Deng et al., 2015; Town et al., 2016; Moreno-Avitia et al., 2017; Biessy et al., 2019) will help to refine the current classification of the *P. chlororaphis* group.

### Main Traits Involved in Biocontrol by *P. chlororaphis*

These aerobic, Gram-negative bacteria are associated with soil and plant roots (González-Sánchez et al., 2010; Calderón et al., 2015; Vida et al., 2017a). Typically, this species possesses plant-colonizing and antagonistic activities against soil-borne plant pathogens. Products from secondary metabolism usually mediate antagonism, and can be regulated by the GacS-GacA two component regulatory system. GacS-GacA system governs a complex signal transduction pathway, involving regulatory RNAs and translational repression (Yan et al., 2018; Jahanshah et al., 2019). Simultaneously, Quorum Sensing (QS) is a regulatory systems which is involved in the general biology performance of *P. chlororaphis*, including biofilm formation, antifungal production or exoenzyme secretion. QS is a mechanism of intercellular signaling that makes the bacterial population to act co-ordinately, based in the secretion of diffusible signal molecules (mainly acyl homoserine lactones, or AHL; Venturi, 2006). The use of OMICs and functional studies have revealed a more complex scenario, where the presence of several QS systems can coexist inside the same bacterial cell (Morohoshi et al., 2017), but also the participation of secondary metabolites (such as the antifungals phenazines and/or the resorcinol-related compounds) in final QS regulation (Selin et al., 2010; Brameyer et al., 2015).

Recent reports using OMICs techniques, have allowed a more comprehensive understanding of the potential weaponry that *P. chlororaphis* group could uses to interact with the root plant. For example, presence of different antimicrobial and insecticidal compounds, cyclic peptides, siderophores, bacteriocins, molecules involved in beneficial plant-bacteria interactions, secretions systems, antibacterial proteins, etc., (Loper et al., 2012; Chen et al., 2015; Biessy et al., 2019). Below, the most relevant are summarized (Table 1).

**Table 1:** Summary of main compounds produced by *Pseudomonas chlororaphis* subspecies with beneficial effects in plant pathogen control.

Compound	Target/beneficial effect	Subspecies^1^	Reference strain	References
**Antibiotics**				
Phenazine 1-carboxamide	Antifungal redox-active antibiotic	Pa, Pe, Pc, Pp	PCL1391	Hernández et al., 2004
Phenazine 1-carboxylic acid	Antifungal redox-active antibiotic	Pc, Pp	PCL1391	Chin-A-Woeng et al., 1998
2-hydroxy phenazine 1-carboxylic acid	Fungistatic and bacteriostatic	Pa, Pe, Pc	GP72	Liu et al., 2016
Pyrrolnitrin	Antifungal compound	Pa, Pe, Pc,	PA23	Nandi et al., 2015
2-hexyl, 5-propylresorcinol	Antifungal compound and signal molecule	Pa, Pe, Pc	PCL1606	Cazorla et al., 2006
2,4 Diacetylphloroglucinol	Membrane damage, distribution of mitochondria electron transport chain and inhibition of V-ATPase activity. Antifungal	Pc	UFB2	Deng et al., 2015
Rhizoxin	Antifungal	Pc	MA 342	Loper et al., 2008
**Insecticidal compounds**				
Cyclic peptides	Insecticidal, surfactant and antagonistic activity	Pc	PCL1391	Flury et al., 2017
Fit toxin	Insecticidal activity	Pc, Pe, Pp	PCL1606	Flury et al., 2016
**Siderophores**				
Pyoverdine	Fe chelation and competition	Pa, Pe, Pc	D-TR133	Barelmann et al., 2003
Achromobactine	Fe chelation and competition	Pa, Pe, Pc	PCL1606	Calderón et al., 2015
Hemophore	Fe chelation	Pp	PCL1607	Biessy et al., 2019
**Enzymes and hormones**				
Chitinase	Chitin hydrolysis enzyme and antifungal	Pc, Pe, Pp	PCL1391	Flury et al., 2016
Protease	Protein hydrolysis enzyme and antifungal	Pa	M71	Raio et al., 2017
Phosphatase	Phosphorus solubilization enzyme	Pc	SZY6	Ahemad, 2015
ACC deaminase	Plant growth promotion	Pa, Pe, Pc, Pp	6G5	Glick, 2014
PQQ	Plant growth promotion	Pa, Pe, Pc	B23	Nishiyama et al., 1991
IAA	Plant growth promotion	Pa, Pe, Pc	O6	Kang et al., 2006
**Volatile**				
2,3 butanediol	Elicite plant resistance	Pa, Pe, Pc	O6	Han et al., 2006
Hydrogen cyanide	Metalloenzymes inhibitor and antifungal	Pa, Pc, Pe, Pp	PA23	Nandi et al., 2015
**Hormones**				
Indol acetic acid	Plant growth promotion	Pa, Pc, Pe, Pp	O6	Kang et al., 2006


*Reference strains published are included. ^*1*^Pa: *P. chlororaphis* subsp. *aurantiaca*; Pe: *P. chlororaphis* subsp. *aureofaciens*; Pc: *P. chlororaphis* subsp. *chlororaphis*; Pp: *P. chlororaphis* subsp. *Piscium.**

Phenazines are among the most copious secondary metabolites produced by fluorescent pseudomonads, and phenazine-producing microorganisms represent a ubiquitous group of antibiotic-producing bacteria in the environment (Chin-A-Woeng et al., 2000; Mavrodi et al., 2013). Phenazine compounds are redox-active nitrogen-containing heterocyclic molecules and its beneficial role on plant biology is not limited to antibiosis against phytopathogenic microbes (Pierson and Pierson, 2010; Biessy and Filion, 2018; Biessy et al., 2019). Additional effects have been shown for this compound such as triggering induced systemic resistance in plants, reducing the expression of key pathogenicity-related genes of the phytopathogen, or its involvement in the root persistence (Biessy and Filion, 2018). In relation to the bacterial interaction with the plant root, phenazines can be crucial for biofilm formation (Selin et al., 2010). An extensive colonization of the rhizosphere is a prerequisite in efficient disease suppression by preventing pathogen form access to the root (Lugtenberg and Kamilova, 2009). The involvement of phenazines on root colonization has been strengthened because some phenazine compounds could be terminal signaling factors in the QS network of some bacteria, and are directly involved in biofilm formation on biotic surfaces (Dietrich et al., 2006; Selin et al., 2012).

Pyrrolnitrin and the volatile compound hydrogen cyanide, are also among the additional antifungal compounds typically produced by *P. chlororaphis* strains. Pyrrolnitrin is considered a key compound for fungal biocontrol (Hill et al., 1994) and is becoming even more relevant than phenazines extending its action to eukaryotic organisms (Nandi et al., 2015; Huang et al., 2018). The same observation can be applied to the volatile compound hydrogen cyanide, which also has a broad spectrum of prokaryotic and eukaryotic targets (Nandi et al., 2017; Kang et al., 2018). The biological importance of this broad spectrum of both active compounds would be related to its typical environmental persistence, for example, allowing them to escape from predation (Nandi et al., 2017). Related to the insecticidal activity of this bacterial species, the most studied virulence factor against insects is the Fit toxin, which is similar to Mcf1 of the entomopathogenic bacterium *Photorhabdus luminescens* (Ruffner et al., 2015). Fit mutants of *P. chlororaphis* PCL1391 further showed reduced virulence, and the residual toxicity could be assigned to the wide range of other antimicrobial compounds produced by *P. chlororaphis* (previously listed) or cyclic lipopetides (Flury et al., 2017).

About Clps, these compounds can be involved in many biological functions, such as motility, biofilm formation, protection against predators and antagonism (De Souza et al., 2003; Raaijmakers et al., 2010). Clps produced by plants-beneficial bacteria were found to induce plant resistance and to contribute to plant protection against root pathogenic fungi (Olorunleke et al., 2015). But interestingly, Clps were demonstrated to be further insect pathogenicity factor in *P. chlororaphis* strains (Flury et al., 2016, 2017).

The production of exoenzymes has also been described to have a role in biocontrol activity (Haran et al., 1996). Enzymes such as chitinases, lipases or proteases have a broad distribution among the soil bacterial community and are probably related to general metabolism, but also inhibit the pathogen (degrading some cell structures) and stimulate plant growth by providing additional resources from the degradative activity (Vida et al., 2017a). Remarkably, *P. chlororaphis* strains can produce 1-aminocyclopropane-1-carboxylate (ACC) deaminase (Nadeem et al., 2007), which is an enzyme produced by plant-associated bacteria that decrease the ethylene levels and protect the plant from its effect, which results in a general beneficial activity (Glick, 2014). In addition, the production of the biofertilizer hormone indole-3-acetic acid (IAA) has also been reported for *P. chlororaphis* strains (Dimkpa et al., 2012), and its production is important in microbe-microbe and microbe-plant signaling, and can also results in an promotion of plant growth (Kang et al., 2006).

Other compounds can also have an important role for *P. chlororaphis*, such as the production of siderophores, which can be considered as a general beneficial activity, at least, for all the soil-related *Pseudomonas* spp. (Zhang and Rainey, 2013). These molecules are secondary metabolites involved in iron quelation. The most known is pyoverdine, a water-soluble fluorescent pigment produced by fluorescent *Pseudomonas* species (Barelmann et al., 2003). However, the recent comparative genomic studies of *P. chlororaphis* genomes, revealed the putative presence of various secondary siderophores, such as achromobactine and hemophore (Biessy et al., 2019).

## The Beneficial Rhizobacterium *Pseudomonas chlororaphis* PCL1606 (PcPCL1606) as a Model

In order to find potential bacterial biocontrol agents against the avocado white root rot caused by *Rosellinia necatrix*, a collection of bacterial isolates belonging to the genera *Bacillus* and *Pseudomonas* were isolated from avocado rhizosphere (Cazorla et al., 2006, 2007; Pliego et al., 2011). Interestingly, a number of *P. chlororaphis* were consistently isolated from avocado roots (Cazorla et al., 2006). The management of this crop could enhance this presence on avocado roots of *P. chlororaphis* isolates, since it has been reported that application of organic amendments can enhance the presence of specific groups of beneficial microbes, including antagonistic *P. chlororaphis* (Vida et al., 2016).

### PcPCL1606 as a Biological Control Agent

**FIGURE 1:**
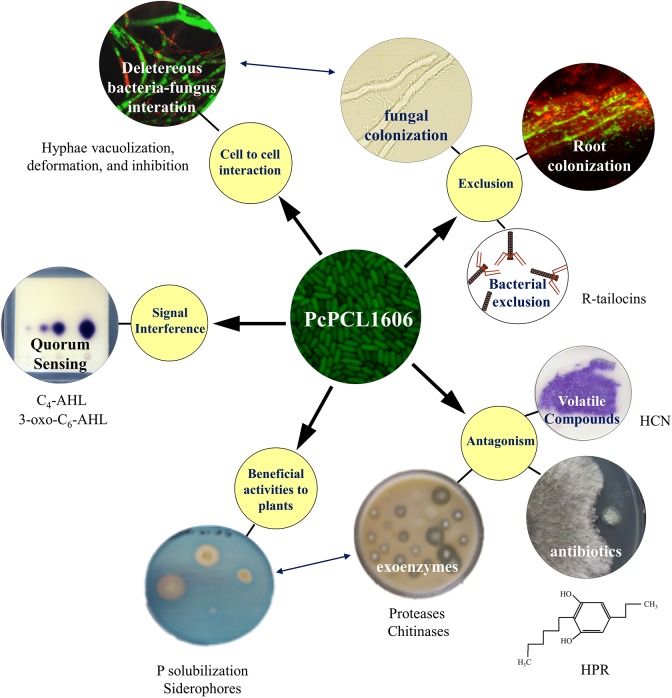
Current knowledge of the strategies displayed during the multitrophic interactions with plants and fungal pathogens by the model rhizobacterial biocontrol strain PcPCL1606. The main strategies involved in the interaction (yellow circles) included cell-to-cell interaction, beneficial interaction with plants, exclusion, antibiotic production and signal interference. The different strategies can enhance a second one (double-ended blue arrows).

Nearly all the *P. chlororaphis* isolated from avocado roots were antagonistic and produced a broad range of antimicrobials including phenazines. Among them, the strain PcPCL1606 do not produce phenazines; otherwise produce proteases, lipases and the antifungal metabolite 2-hexyl 5-propylresorcinol (HPR; Figure 1). Another unusual characteristic of this strain is the absence of plant growth promotion in the assayed plant models; however, siderophore production and phosphorous solubilization were detected (among other PGPR-related traits; Vida et al., 2017a). This strain displayed strong antagonism to many phytopathogenic fungi and showed biocontrol of crown and root rot of tomato, caused by *Fusarium oxysporum* f. sp. *radicis-lycopersici* and avocado white root, caused by *R. necatrix* (Cazorla et al., 2006; González-Sánchez et al., 2013). Effectiveness of biocontrol was directly related to the compound HPR (Cazorla et al., 2006; Calderón et al., 2013). HPR production was led by three biosynthetic genes located in a cluster (*dar*A, *dar*B, and *dar*C) followed by two independent regulatory genes (*dar*S and *dar*R; Nowak-Thompson et al., 2003; Calderón et al., 2013). Further experiments revealed that HPR production was also under transcriptional regulation of the GacS-GacA two-component regulatory system, as previously described for other antifungal antibiotics (Haas and Keel, 2003), and also modulated by different growth parameters such as temperature, pH and the presence of salts in the medium (Calderón et al., 2014a).

### Main Features of PcPCL1606 Involved in Pathogen and Plant Interaction

PcPCL1606 showed strong antifungal activity (Figure 1), and HPR production was the main determinant in the antagonistic and biocontrol phenotypes (Calderón et al., 2013). In addition to HPR, other antifungals can be produced by PcPCL1606, such as pyrrolnitrin (PRN) or hydrogen cyanide (HCN), as well as several exoenzymes such as proteases, chitinases or phosphatases (Vida et al., 2017a). Nevertheless, HPR is more than a powerful compound against pathogenic fungi in the soil and could have additional roles. It has been reported that some alkylresorcinols (to which the compound HPR belongs) can behave as quorum sensing-like signal molecules in the genus *Photorhabdus* (Brameyer et al., 2015), and for this, could have a similar role in HPR-producing *P. chlororaphis* strains. Thus, additional HPR-dependent traits, which are different from antagonism, could have an essential role in the beneficial effects of PcPCL1606 on the plant, such as the root colonization or the biofilm formation (Calderón et al., 2014b, 2019).

Related to the possibility to physically exclude the pathogen from the plant root habitat (Figure 1), biological processes, such as biofilm formation or chemotaxis, are crucial for the PcPCL1606. PcPCL1606 is strongly attracted to the avocado root exudates by chemotactic processes (Polonio et al., 2017). As a result of this attraction, PcPCL1606 efficiently colonizes avocado roots (González-Sánchez et al., 2010) and can be found forming a biofilm on avocado root surfaces, located in the same area where *R. necatrix* can be found during the early stages of infection (Calderón et al., 2014b). Moreover, two bacteriocins (R-tailocins 1 and 2), recently described in PcPCL1606 would contribute to better competition against other rhizosphere-associated bacteria (Dorosky et al., 2017). However, PcPCL1606 bacterial cells also displayed a direct chemotaxis to fungal exudates and finally showed a direct contact with the fungal hyphae of *R. necatrix*. This cell-to-cell contact causes an increase in stress symptoms on the hyphae, among others, by the direct release of antifungal substances, which lead to an accelerated ageing process in the hyphae and hyphal death (Calderón et al., 2014b; Moore-Landecker, 1996). Moreover, the root colonization ability and biofilm formation of the wild-type strain was also related to HPR production, and the absence of HPR resulted in reduced root colonization levels and no biofilm formation by PcPCL1606 (Calderón et al., 2014b, 2019).

To obtain insight into the features of PcPCL1606, its complete genome sequencing was completed. Phylogenetic studies clustered this strain into the *P. chlororaphis* clade which is placed into the fluorescent *Pseudomonas* complex, however, as previously mentioned, PcPCL1606 it is not a typical *P. chlororaphis* strain (Biessy et al., 2019). Thus, phylogenetic analysis revealed clear differences with the genomes of other biocontrol *P. chlororaphis*, such as PcPCL1601 or PcPCL1607, also isolated from avocado root (Calderón et al., 2015; Vida et al., 2017b; Biessy et al., 2019). Analysis of PcPCL1606 genome confirmed a lack of phenazine biosynthetic genes, cyclic lipopeptides that are related to the surfactant and insecticidal properties, which are typical for *P. chlororaphis*x (Raaijmakers et al., 2006). However, PcPCL1606 exhibits a complete Fit toxin (*fit*) cluster (Calderón et al., 2015).

## Futures Projects and Research

The future of *P. chlororaphis* as biocontrol agent is very promising. *P. chlororaphis* is ubiquitous in the environment, lacks known toxic or allergenic properties, and has a history of safe use in agriculture and in food and feed crops. *P. chlororaphis* is considered non-pathogenic to humans, wildlife or the environment according to the United States Environmental Protection Agency (EPA), and commercial products based on *P. chlororaphis* strains are already available. For example, Cedomon^®^ (*P. chlororaphis*, BioAgri AB, Sweden), Spot-Less^®^ (*P. aureofaciens* Tx-1, Turf Science Laboratories, Carlsbad, United States) or AtEze^®^ (*P. chlororaphis* 63-28, Turf Science Laboratories, Carlsbad, United States) are based on *P. chlororaphis* strains, but many other products are already present in the market based on other *Pseudomonas* spp. These facts pointed out to a promising future for the use of biocontrol agents belonging to the specie *P. chlororaphis*.

Regarding the model bacterium PcPCL1606, studies revealed that PcPCL1606, as well as other *P. chlororaphis* isolates from avocado roots, displayed high persistence and reached a population density that was enough to reduce disease (González-Sánchez et al., 2013). Under commercial greenhouse conditions, applications of PcPCL1606 cells resulted in biocontrol against *R. necatrix*. Moreover, some other *P. chlororaphis* isolates from avocado roots, that have different beneficial traits (such as phenazine production or plant growth promotion), could also provide plant protection. These finding suggest that a promising approach to improve *P. chlororaphis* based biocontrol would be to develop consortia which combine strains with complementary traits resulting in more stable or even enhanced beneficial effects on plants.

## Author Contributions

EA and FC designed the review content. EA, ST, CV, AV, and FC wrote the manuscript. All authors read and approved the final manuscript.

## Conflict of Interest Statement

The authors declare that the research was conducted in the absence of any commercial or financial relationships that could be construed as a potential conflict of interest.

## References

[B1] Ahemad M. (2015). Phosphate-solubilizing bacteria-assisted phytoremediation of metalliferous soils: a review. *3 Biotech* 5 111–121. 10.1007/s13205-014-0206-0 28324572 PMC4362741

[B2] Babalola O. O. (2010). Beneficial bacteria of agricultural importance. *Biotechnol. Lett.* 32 1559–1570. 10.1007/s10529-010-0347-0 20635120

[B3] Barelmann I. Fernández D. U. Budzikiewicz H. Meyer J. M. (2003). The pyoverdine from *Pseudomonas chlororaphis* D-TR133 showing mutual acceptance with the pyoverdine of *Pseudomonas fluorescens* CHA0. *Biometals* 16 263–270. 10.1023/A:1020615830765 12572684

[B4] Berendsen R. L. Vismans G. Yu K. Song Y. de Jonge R. Burgman W. P. (2018). Disease-induced assemblage of a plant-beneficial bacterial consortium. *ISME J.* 12 1496–1507. 10.1038/s41396-018-0093-1 29520025 PMC5956071

[B5] Berg G. (2009). Plant-microbe interactions promoting plant growth and health: perspectives for controlled use of microorganisms in agriculture. *Appl. Microbiol. Biotechnol.* 84 11–18. 10.1128/AEM.68.7.3328-3338.2002 19568745

[B6] Berg G. Roskot N. Steidle A. Eberl L. Zock A. Smalla K. (2002). Plant-dependent genotypic and phenotypic diversity of antagonistic rhizobacteria isolated from different *Verticillium* host plants. *Appl. Environ. Microbiol.* 68 3328–3338. 10.1128/AEM.68.7.3328-3338.2002 12089011 PMC126805

[B7] Bergsma-Vlami M. Prins M. E. Raaijmakers J. M. (2005). Influence of plant species on population dynamics, genotypic diversity and antibiotic production in the rhizosphere by indigenous *Pseudomonas* spp. *FEMS Microbiol. Ecol.* 52 59–69. 10.1016/j.femsec.2004.10.007 16329893

[B8] Biessy A. Filion M. (2018). Phenazines in plant-beneficial *Pseudomonas* spp.: biosynthesis, regulation, function and genomics. *Environ. Microbiol.* 20 3905–3917. 10.1111/1462-2920.14395 30159978

[B9] Biessy A. Novinscak A. Blom J. Léger G. Thomashow L. Cazorla F. M. (2019). Diversity of phytobeneficial traits revealed by whole-genome analysis of worldwide-isolated phenazine-producing *Pseudomonas* spp. *Environ. Microbiol.* 21 437–455. 10.1111/1462-2920.14476 30421490

[B10] Bloemberg G. V. Lugtenberg B. J. (2001). Molecular basis of plant growth promotion and biocontrol by rhizobacteria. *Curr. Opin. Plant Biol.* 4 343–350. 10.1016/S1369-5266(00)00183-7 11418345

[B11] Brameyer S. Kresovic D. Bode H. B. Heermann R. (2015). Dialkylresorcinols as bacterial signaling molecules. *Proc. Natl. Acad. Sci. U.S.A.* 112 572–577. 10.1073/pnas.1417685112 25550519 PMC4299209

[B12] Burr S. E. Gobeli S. Kuhnert P. Goldschmidt-Clermont E. Frey J. (2010). Pseudomonas chlororaphis subsp. Piscium subsp. nov., isolated from freshwater fish. *Int. J. Syst. Evol. Microbiol.* 60 2753–2757. 10.1099/ijs.0.011692-0 20061493

[B13] Calderón C. E. Carrión V. J. de Vicente A. Cazorla F. M. (2014a). *dar*R and *dar*S are regulatory genes that modulate 2-hexyl, 5-propyl resorcinol transcription in *Pseudomonas chlororaphis* PCL1606. *Microbiology* 160 2670–2680. 10.1099/mic0.082677-0 25234473

[B14] Calderón C. E. de Vicente A. Cazorla F. M. (2014b). Role of 2-hexyl, 5-propyl resorcinol production by *Pseudomonas chlororaphis* PCL1606 in the multitrophic interactions in the avocado rhizosphere during the biocontrol process. *FEMS Microbiol. Ecol.* 89 20–31. 10.111/1574-6941.12319 24641321

[B15] Calderón C. E. Pérez-García A. de Vicente A. Cazorla F. M. (2013). The *dar* genes of *Pseudomonas chlororaphis* PCL1606 are crucial for biocontrol activity via production of the antifungal compound 2-hexyl, 5-propyl resorcinol. *Mol. Plant Microbe Interact.* 26 554–565. 10.1094/MPMI-01-13-0012-R 23547906

[B16] Calderón C. E. Ramos C. de Vicente A. Cazorla F. M. (2015). Comparative genomic analysis of *Pseudomonas chlororaphis* PCL1606 reveals new insight into antifungal compounds involved in biocontrol. *Mol. Plant Microbe Interact.* 28 249–260. 10.1094/MPMI/10-14-0326-FI 25679537

[B17] Calderón C. E. Tienda S. Heredia-Ponce Z. Arrebola E. Cárcamo-Oyarce G. Eberl L. (2019). The compound 2-hexyl, 5-propyl resorcinol has a key role in biofilm formation by the biocontrol rhizobacterium *Pseudomonas chlororaphis* PCL1606. *Front. Microbiol.* 10:396. 10.3389/fmicb.2019.00396 30873149 PMC6403133

[B18] Cazorla F. M. Duckett S. D. Bergström E. T. Odijk R. Lugtenberg B. J. J. Thomas-Oates J. E. (2006). Biocontrol of avocado dematophora root rot by antagonistic *Pseudomonas fluorescens* PCL1606 correlates with the production of 2-hexyl, 5-propyl resorcinol. *Mol. Plant Microbe Interact.* 19 418–428. 10.1094/MPMI-19-0418 16610745

[B19] Cazorla F. M. Romero D. Pérez-García A. Lugtenberg B. J. J. de Vicente A. Bloemberg G. (2007). Isolation and characterization of antagonistic *Bacillus subtillis* strains from the avocado rhizoplane displaying biocontrol activity. *J. Appl. Microbiol.* 103 1950–1959. 10.111/j.1365-2672.2007.03433.x 17953605

[B20] Chen Y. Shen X. Peng H. Hu H. Wang W. Zhang X. (2015). Comparative genomic analysis and phenazine production of *Pseudomonas chlororaphis*, a plant growth-promoting rhizobacterium. *Gemon. Data* 4 33–42. 10.1010/j.gdata.2015.01.006 26484173 PMC4535895

[B21] Chin-A-Woeng T. F. C. Bloemberg G. V. Mulders I. H. Dekkers L. C. Lugtenberg B. J. (2000). Root colonization by phenazine-1-carboxamide-producing bacterium *Pseudomonas chlororaphis* PCL1391 is essential for biocontrol of tomato foot and root rot. *Mol. Plant Microbe Interact.* 13 1340–1345. 10.1094/MPMI.2000.13.12.1340 11106026

[B22] Chin-A-Woeng T. F. C. Bloemberg G. V. van der Bij A. J. van der Drift K. M. G. M. Schripsema J. Kroon B. (1998). Biocontrol by phenazine-1-carboxamide-producing *Pseudomonas chlororaphis* PCL1391 of tomato root rot caused by *Fusarium oxysporum* f. sp. *radicis-lycopersici*. *Mol. Plant-Microbe Interact.* 11 1069–1077. 10.1094/MPMI.1998.11.11.1069

[B23] De Souza J. T. De Boer M. De Waard P. Van Beek T. A. Raaijmakers J. M. (2003). Biochemical, genetic, and zoosporicidal properties of cyclic lipopeptide surfactants produced by *Pseudomonas fluorescens*. *Appl. Environ. Microbiol.* 69 7161–7172. 10.1128/AEM.69.127161-7172.2003 14660362 PMC309978

[B24] Deng P. Wang X. Baird S. M. Lu S. E. (2015). Complete genome of *Pseudomonas chlororaphis* strain UFB2, a soil bacterium with antibacterial activity against bacterial canker pathogen of tomato. *Stand. Genomic Sci.* 10:117. 10.1186/s40793-015-0106-x 26634018 PMC4667424

[B25] Dietrich L. E. P. Price-Whelan A. Petersen A. Whiteley M. Newman D. K. (2006). The phenacine pyocyanin is a terminal signaling factor in the quorum-sensing network of *Pseudomonas aeruginosa*. *Mol. Microbiol.* 61 1308–1321. 10.1111/j.1365-2958.2006.05306.x 16879411

[B26] Dimkpa C. O. Zeng J. McLean J. E. Britt D. W. Zhan J. Anderson A. J. (2012). Pathway in the plant-beneficial bacterium *Pseudomonas chlororaphis* O6 is inhibited by ZnO nanoparticles but enhanced by CuO nanoparticles. *Appl. Environ. Microbiol.* 78 1404–1410. 10.1128/AEM.07424-11 22210218 PMC3294495

[B27] Dorosky R. J. Yu J. M. Pierson L. S. III Pierson E. A. (2017). *Pseudomonas chlororaphis* produces two distinct R-tailocins that contribute to bacterial competition in biofilm and on roots. *Appl. Environ. Microbiol.* 83:e706–17. 10.1128/AEM.00706-17 28526791 PMC5514687

[B28] Flury P. Aellen N. Ruffner B. Péchy-Tarr M. Fataar S. Metla Z. (2016). Insect pathogenicity in plant-beneficial pseudomonads: phylogenetic distribution and comparative genomics. *ISME* 10 2527–2542. 10.1038/ismej.2016.5 26894448 PMC5030700

[B29] Flury P. Vesga P. Péchy-Tarr M. Aellen N. Dennert F. Hofer N. (2017). Antimicrobial and insecticidal: cyclic lipopeptides and hydrogen cyanide produced by plant-beneficial *Pseudomonas* strains CHA0, CMR12a, and PCL1391 contribute to insect killing. *Front. Microbiol.* 8:100. 10.3389/fmicb2017.00100 28217113 PMC5289993

[B30] Ganeshan G. Kumar A. M. (2005). *Pseudomonas fluorescens* a potential bacterial antagonist to control plant diseases. *J. Plant Interact.* 13 123–134. 10.1080/17429140600907043

[B31] Garrido-Sanz D. Arrebola E. Martínez-Granero F. García-Méndez S. Muriel C. Blanco-Romero E. (2017). Classification of isolates from the *Pseudomonas fluorescens* complex into phylogenomic groups based in group-specific markers. *Front. Microbiol.* 8:413. 10.3389/fmicb.2017.00413 28360897 PMC5350142

[B32] Glick B. R. (2014). Bacteria with ACC deaminase can promote plant growth and help to feed the world. *Microbiol. Res.* 169 30–39. 10.1016/j.micres.2013.09.009 24095256

[B33] González-Sánchez M. A. de Vicente A. Pérez-García A. Pérez-Jiménez R. Romero D. Cazorla F. M. (2013). Evaluation of the effectiveness of biocontrol bacteria against avocado white root rot occurring under commercial greenhouse plant production conditions. *Biol. Control* 67 94–100. 10.1016/j.biocontrol.2013.08.009

[B34] González-Sánchez M. A. Pérez-Jiménez R. M. Pliego C. Ramos C. de Vicente A. Cazorla F. M. (2010). Biocontrol bacteria selected by a direct plant protection strategy against avocado white root rot show antagonism as a prevalent trait. *J. Appl. Microbiol.* 109 65–78. 10.1111/j.1365-2672.2009.04628.x 19961545

[B35] Haas D. Défago G. (2005). Biological control of soil-borne pathogens by fluorescent pseudomonads. *Nat. Rev. Microbiol.* 3 307–319. 10.1038/nrmicro1129 15759041

[B36] Haas D. Keel C. (2003). Regulation of antibiotic production in root-colonizing *Pseudomonas* spp. and relevance for biological control of plant disease. *Annu. Rev. Phytopathol.* 41 117–153. 10.1146/annurev.phyto.41.052002.095656 12730389

[B37] Han S. H. Lee S. J. Moon J. H. Park K. H. Yang K. Y. Cho B. H. (2006). GacS-dependent production of 2R, 3R-butanediol by *Pseudomonas chlororaphis* O6 is a mayor determinant for eliciting systemic resistance against *Erwinia carotovora* but not against *Pseudomonas syringae* pv. Tabaci in tobacco. *Mol. Plant Microbe Interact.* 19 924–930. 10.1094/MPMI-19-0924 16903358

[B38] Haran S. Schickler H. Chet I. (1996). Molecular mechanisms of lytic enzymes involved in the biocontrol activity of *Trichoderma harzianum*. *Microbiology* 142 2321–2331. 10.1099/00221287-142-9-2321

[B39] Hernández M. E. Kappler A. Newman D. K. (2004). Phenazines and other redox-active antibiotics promote microbial mineral reduction. *Appl. Environ. Microbiol.* 70 921–928. 10.1128/AEM.70.2.921-928.2004 14766572 PMC348881

[B40] Hilario E. Buckley T. R. Young J. M. (2004). Improved resolution on the phylogenetic relationships among *Pseudomonas* by the combined analysis of *atp*D, *car*A, *rec*A and 16S rDNA. A. van Leeuw. *J. Microb.* 86 51–64. 10.1023/B.ANTO.0000024910.57117.16 15103237

[B41] Hill D. S. Stein J. I. Torkewitz N. R. Morse A. M. Howell C. R. Pachlatko J. P. (1994). Cloning of genes involved in the synthesis of pyrrolnitrin from *Pseudomonas fluorescens* and role of pyrrolnitrin synthesis in biological control of plant disease. *Appl. Environ. Microbiol.* 60 78–85. 16349167 10.1128/aem.60.1.78-85.1994 PMC201272

[B42] Huang R. Feng Z. Chi X. Sun X. Lu Y. Zhang B. (2018). Pyrrolnitrin is more essential than phenazines for *Pseudomonas chlororaphis* G05 in its suppression of *Fusarium graminearum*. *Microbiol. Res.* 215 55–64. 10.1016/j.micres.2018.06.008 30172309

[B43] Jahanshah G. Yan Q. Gerhardt H. Pataj Z. Lämnerhofer M. Pianet I. (2019). Dicovery of the cyclic lipopeptide gacamide A by genome mining and repair of the defective GacA regulator in *Pseudomonas fluorescens* Pf0-1. *J. Nat. Prod.* 82 301–308. 10.1021/acs.jnatprod.8b00747 30666877

[B44] Kang B. R. Anderson A. J. Kim Y. C. (2018). Hydrogen cyanide produced by *Pseudomonas chlororaphis* O6 exhibits nematicidal activity against *Meloidogyne hapla*. *Plant Pathol. J.* 34 35–43. 10.5423/PPJ.OA.06.2017.0115 29422786 PMC5796748

[B45] Kang B. R. Yang K. Y. Cho B. H. Han T. H. Kim I. S. Lee M. C. (2006). Production of indole-3-acetic acid in the plant-beneficial strain *Pseudomonas chlororaphis* O6 is negatively regulated by the global sensor kinase GacS. *Curr. Microbiol.* 52 473–476. 10.1007/s00284-005-0427-x 16732458

[B46] Liu K. Hu H. Wang W. Zhang X. (2016). Genetic engineering of *Pseudomonas chlororaphis* GP72 for the enhanced production of 2-hydroxyphenazine. *Microb. Cell Fact.* 15:131. 10.1186/s12934-016-0529-0 27470070 PMC4965901

[B47] Loper J. E. Hassan K. A. Mavrodi D. V. Davis E. W. I. I. Lim C. K. Shaffer B. T. (2012). Comparative genomics of plant-associated *Pseudomonas* spp.: insight into diversity and inheritance of traits involved in multitrophic interactions. *PLoS Genet.* 8:e1002784. 10.1371/journal.pgen.1002784 22792073 PMC3390384

[B48] Loper J. E. Henkels M. D. Shaffer B. T. Valeriote F. A. Gross H. (2008). Isolation and identification of rhizoxin analogs from *Pseudomonas fluorescens* Pf-5 by using a genomic mining strategy. *Appl. Environ. Microbiol.* 74 3085–3093. 10.1128/AEM.02848-07 18344330 PMC2394923

[B49] Lugtenberg B. J. J. Dekkers L. C. (1999). What makes *Pseudomonas* bacteria rhizosphere competent? *Environ. Microbiol.* 1 9–13. 10.1046/j.1462-2920.1999.00005.x 11207713

[B50] Lugtenberg B. J. J. Kamilova F. (2009). Plant-growth-promoting rhizobacteria. *Annu. Rev. Microbiol.* 63 541–556. 10.1146/annurev.micro.62.081307.162918 19575558

[B51] Mavrodi D. V. Parejko J. A. Mavrodi O. V. Kwak Y. S. Weller D. M. Blankenfeldt W. (2013). Recent insights into the diversity, frequency and ecological roles of phenazines in fluorescent *Pseudomonas* spp. *Environ. Microbiol.* 15 675–686. 10.1111/j.1462-2920.2012.02846.x 22882648

[B52] Mazzola M. Funnell D. L. Raaijmakers J. M. (2004). Wheat cultivar-specific selection of 2,4-diacetylphloroglucinol-producing fluorescent *Pseudomonas* species from resident soil populations. *Microbiol. Ecol.* 48 338–348. 10.1007/s00248-003-1067-y 15692854

[B53] McSpadden B. B. (2007). Diversity and ecology of biocontrol *Pseudomonas* spp. in agricultural systems. *Phytopathology* 97 221–226. 10.1094/PHYTO-97-2-0221 18944378

[B54] Mercado-Blanco J. Bakker P. A. H. M. (2007). Interactions between plants and beneficial *Pseudomonas* spp.: exploiting bacterial traits from crop protection. *Antonie Van Leeuw.* 92 367–389. 10.1007/s10482-007-9167-1 17588129

[B55] Moore-Landecker E. (1996). *Fundamentals of the Fungi*, 4th Edn. Saddle River, NJ: Prentice Hall.

[B56] Moreno-Avitia F. Lozano L. Utrilla J. Bolívar F. Escalante A. (2017). Draft genome sequence of *Pseudomonas chlororaphis* ATCC 9446, a nonpathogenic bacterium with bioremediation and industrial potential. *Genome Announc.* 5:e474–17. 10.1128/genomeA.00474-17 28596401 PMC5465620

[B57] Morohoshi T. Yamaguchi T. Xie X. Wang W. Z. Takeuchi K. Someya N. (2017). Complete genome sequence of *Pseudomonas chlororaphis* subsp. Auranthiaca reveals a triplicate quorum-sensing mechanism for regulation of phenazine production. *Microbes Environ.* 32 47–53. 10.1264/jsme2.ME16162 28239068 PMC5371074

[B58] Nadeem S. M. Zahir Z. A. Naveed M. Arshad M. (2007). Preliminary investigation on inducing salt tolerance in maize through inoculation with rhizobacteria containing ACC deaminase activity. *Can. J. Microbiol.* 53 1141–1149. 10.1139/W07-081 18026206

[B59] Nandi M. Selin C. Brassinga A. K. C. Belmonte M. F. Fernando W. G. D. Loewen P. C. (2015). Pyrronitrin and hydrogen cyanide production by *Pseudomonas chlororaphis* PA23 exhibits nematicidal and repellent activity against *Caenorhabditis elegans*. *PLoS One* 10:e0123184. 10.1371/journaol.pone.0123184 25901993 PMC4406715

[B60] Nandi M. Selin C. Brawerman G. Fernando W. G. D. de Kievit T. (2017). Hydrogen cyanide, which contributes to *Pseudomonas chlororaphis* strain PA23 biocontrol, is upregulated in the presence of glycine. *Biol. Control* 108 47–54. 10.1016/j.biocontrol.2017.02.008

[B61] Nishiyama M. Horinouchi S. Kobayashi M. Nagasawa T. Yamada H. Beppu T. (1991). Cloning and characterization of genes responsible for metabolism of nitrile compounds from *Pseudomonas chlororaphis* B23. *J. Bacteriol.* 173 2465–2472. 10.1128/jb.173.8.2465-2472.1991 2013568 PMC207809

[B62] Nowak-Thompson B. Philip E. Hammer D. Hill D. S. Staffords J. Torkewitz N. (2003). 2, 5-diakylresorcinol biosynthesis in *Pseudomonas aurantiaca*: novel head-to-head condensation of two fatty acid-derived precursors. *J. Bacteriol.* 185 860–869. 10.1128/JB.185.3.860-869.2003 12533461 PMC142816

[B63] Olorunleke F. E. Hua G. K. Kieu N. P. Ma Z. Höfte M. (2015). Interplay between orfamides, sessilins and phenazines in the control of rhizoctonia diseases by *Pseudomonas* sp. *CMR*12a. *Environ. Microbiol. Rep.* 7 774–781. 10.1111/1758-2229.12310 26085277

[B64] Ownley B. H. Duffy B. K. Weller D. M. (2003). Identification and manipulation of soil properties to improve the biological control performance of phenazine-producing *Pseudomonas fluorescens*. *Appl. Environ. Microbiol.* 69 3333–3343. 10.1128/AEM.69.6.3333-3343.2003 12788734 PMC161483

[B65] Palleroni N. J. (1984). “Family I. Pseudomonadaceae Winslow, Broadhurst, Buchanan, Krumwiede, Rogers and Smith 1917, 555,” in *Bergey’s Manual of Systematic Bacteriology*, 1st Edn, eds Sneath P. H. A. Mair N. S. Sharpe M. E. Holt J. G. (Baltimore, MD: Williams & Wilkins), 144–218.

[B66] Peix A. Valverde A. Rivas R. Igual J. M. Ramírez-Bahena M. H. Mateos P. F. (2007). Reclassification of *Pseudomonas aurantiaca* as a synonym of *Pseudomonas chlororaphis* and proposal of three subspecies, *P. Chlororaphis subsp. Chlororaphis Subsp*. nov., *P. Chlororaphis subsp*. *Aureofaciens subsp.* nov., comb. nov. and P. chlororaphis subsp. aurantiaca subsp. nov., comb. nov. *Int. J. Syst. Evol. Microbiol.* 57 1286–1290. 10.1099/ijs.0.64621-0 17551044

[B67] Pierson L. S. III Pierson E. A. (2010). Metabolism and function of phenazines in bacteria: impacts on the behavior of bacteria in the environment and biotechnological processes. *Appl. Microbiol. Biotechnol.* 86 1659–1670. 10.1007/s00253-010-2509-3 20352425 PMC2858273

[B68] Pliego C. Ramos C. de Vicente A. Cazorla F. M. (2011). Screening for candidate bacterial biocontrol agents against soilborne fungal plant pathogens. *Plant Soil* 340 505–520. 10.1007/s11104-010-0615-8

[B69] Polonio A. Vida C. de Vicente A. Cazorla F. M. (2017). Impact of motility and chemotaxis features of the rhizobacterium *Pseudomonas chlororaphis* PCL1606 on its biocontrol of avocado white root rot. *Int. Microbiol.* 20 95–104. 10.2436/20.1501.01.289 28617527

[B70] Raaijmakers J. M. de Bruijn I. de Kock M. J. D. (2006). Cyclic lipopeptide production by plant-associated *Pseudomonas* spp.: diversity, activity, biosynthesis, and regulation. *Mol. Plant Microbe Interact.* 19 699–710. 10.1094/MPMI-19-0699 16838783

[B71] Raaijmakers J. M. De Bruijn I. Nybroe O. Ongena M. (2010). Natural functions of lipopeptides from *Bacillus* and *Pseudomonas*: more than surfactants and antibiotics. *FEMS Microbiol. Rev.* 34 1037–1062. 10.1111/j.1574-6976.2010.00221.x 20412310

[B72] Raaijmakers J. M. Paulitz T. C. Steinberg C. Alabouvette C. Moënne-Loccoz Y. (2009). The rhizosphere: a playground and battlefield for soilborne pathogens and beneficial microorganisms. *Plant Soil* 321:20 10.1007/s11104-008-9568-6

[B73] Raio A. Reveglia P. Puopolo G. Cimmino A. Danti R. Evidente A. (2017). Involvement of phenaazine-1-carboxylic acid in the interaction between *Pseudomonas chlororaphis* subsp. Aureofaciens strain M71 and Seiridium cardinal in vivo. *Microbiol. Res.* 199 49–56. 10.1016/j.micres.2017.03.003 28454709

[B74] Ruffner B. Péchy-Tarr M. Höfte M. Bloemberg G. Grunder J. Keel C. (2015). Evolutionary patchwork of an insecticidal toxin shared between plant-associated pseudomonads and the insect pathogens *Photorhabdus* and *Xenorhabdus*. *BMC Genomics* 16:609. 10.1186/s12864-015-1763-2 26275815 PMC4542124

[B75] Selin C. Fernando D. de Kievit T. R. (2012). The Phzl/PhzR quorum-sensing system is required for pyrrolnitrin and phenazine production, and exhibits cross-regulation with RpoS in *Pseudomonas chlororaphis* PA23. *Microbiology* 158 896–907. 10.1099/mic.0.054254-0 22262095

[B76] Selin C. Habibian R. Poritsanos N. Athukorala S. N. Fernando D. de Kievit T. R. (2010). Phenazines are not essential for *Pseudomonas chlororaphis* PA23 biocontrol of *Sclerotinia sclerotiorum*, but do play a role in biofilm formation. *FEMS Microbiol. Ecol.* 71 73–83. 10.1111/j.1574-6941.2009.00792.x 19889032

[B77] Skerman V. B. D. McGoawna V. Sneath P. H. A. (1980). Approved lists of bacterial names. *Int. J. Syst. Bacteriol.* 30 225–420. 10.1099/00207713-30-1-225

[B78] Town J. Audy P. Boyetchko S. M. Dumonceaux T. J. (2016). Genome sequence of *Pseudomonas chlororaphis* strain 189. *Genome Announc.* 4:e581–16. 10.1128/genomeA.00581-16 27340063 PMC4919402

[B79] Venturi V. (2006). Regulation of quorum sensing in *Pseudomonas*. *FEMS Microbiol. Rev.* 30 274–291. 10.1111/j.1574-6976.2005.00012.x 16472307

[B80] Vida C. Bonilla N. de Vicente A. Cazorla F. M. (2016). Microbial profiling of a suppressiveness-induced agricultural soil amended with composted almond shells. *Front. Microbiol.* 7:4. 10.3389/fmicb.2016.00004 26834725 PMC4722121

[B81] Vida C. Cazorla F. M. de Vicente A. (2017a). Characterization of biocontrol bacterial strains isolated from a suppressiveness-induced soil after amendment with composted almond shells. *Res. Microbiol.* 168 583–593. 10.1016/j.resmic.2017.03.007 28373145

[B82] Vida C. de Vicente A. Cazorla F. M. (2017b). Draft genome sequence of the rhizobacterium *Pseudomonas chlororaphis* PCL1606, displaying biocontrol against soilborne phytopatogens. *Genome Announc.* 5:e130–17. 10.1128/genomeA.00130-17 PMC5383896 28385848

[B83] Waksman S. A. Woodruff B. H. (1940). The soil as a source of microorganisms antagonistic to disease-producing bacteria. *J. Bacteriol.* 40 581–600. 16560371 10.1128/jb.40.4.581-600.1940 PMC374661

[B84] Weller D. (2007). *Pseudomonas* biocontrol agents of soilborne pathogens: looking back over 30 years. *Phytopathology* 97 250–256. 10.1094/PHYTO-97-2-0250 18944383

[B85] Yan Q. Lopes L. D. Shaffer B. T. Kidarsa T. A. Vining O. Philmus B. (2018). Secondary metabolism and interspecific competition affect accumulation of spontaneous mutants in the GacS-GacA regulatory system in *Pseudomonas protegens*. *mBio* 9:e1845–17. 10.1128/mBio.01845-17 29339425 PMC5770548

[B86] Zhang X. X. Rainey P. B. (2013). Exploring the sociobiology of pyoverdin-producing *Pseudomonas*. *Evolution* 67 3161–3174. 10.1111/evo.12183 24152000

